# Lectures in the Bellevue Hospital Medical College

**Published:** 1870-09

**Authors:** 


### Lectures in the Bellevue Hospital Medical College.

The Faculty of the Bellevue Medical College have secured the services of Professor James P. White, M. D., of Buffalo, as Lecturer upon Obstetrics and Diseases of Women and Children, and Clinical Midwifery, for the coming session, rendered necessary by the illness of Professor George T. Elliot, M.D., the incumbent of the chair. With the view of not interfering with the course of lectures in Buffalo, arrangements have been made by which Professor White's course will be given in New York, commencing October 13th, to be completed in time to commence his usual course in Buffalo, December 26th.

In making this announcement, we are able, at the same time, to say that Prof. White has already assured the Faculty of the Bellevue Hospital College, that nothing but death could take him permanently from Buffalo, so that his friends may rest satisfied that he will not entertain the idea of permanent removal. He has accepted this position, which, in its bestowment, is as high a compliment to his professional standing and attainments as this country could offer, from regard to the wishes of his friend, Prof. Elliott, and other members of the Bellevue Faculty, with some of whom he has been previously and very pleasantly associated in teaching.

We most heartily congratulate the Bellevue Medical College in obtaining the services of so able and distinguished a teacher. He may go with them, for we know he will "do them good." All we ask is, that they use him carefully and return him safely. He is the "Nestor" of the profession in Western New York.

